# Valproate ameliorates nitroglycerin-induced migraine in trigeminal nucleus caudalis in rats through inhibition of NF-кB

**DOI:** 10.1186/s10194-016-0631-z

**Published:** 2016-05-06

**Authors:** Yuanchao Li, Qin Zhang, Dandan Qi, Li Zhang, Lian Yi, Qianqian Li, Zhongling Zhang

**Affiliations:** Department of Neurology, The First Affiliated Hospital of Harbin Medical University, No. 23 Youzheng Road, Harbin, 150001 People’s Republic of China

**Keywords:** Anti-oxidant, Migraine, NF-кB, Nitroglycerin, Valparoate

## Abstract

**Background:**

As a complex nervous system disease, migraine causes severe healthy and social issues worldwide. Valproate (VPA) is a widely used treatment agent against seizures and bipolar disorder, and its function to alleviate damage due to migraine has also been verified in clinical investigations. However, the mechanism underlying the protective effect of VPA against migraine remains poorly revealed. In the current study, the major purpose was to uncover the mechanism which drove VPA to antagonize migraine.

**Methods:**

Nitroglycerin (NTG) was employed to induce a migraine model in rats and the migraine animals were exposed to treatment of VPA of different doses. Thereafter, the levels of indicators related to oxidative stress were measured and used to evaluate the anti-oxidant potential of VPA. The expression of calcitonin gene-related peptide (CGRP) and c-Fos was also quantified with ELISA and immunohistochemistry, respectively. Western blotting and electrophoretic mobility shift assays (EMSA) were conducted to explore the effect of VPA treatment on NF-кB pathway.

**Results:**

NTG induced the activation of oxidative stress and led to migraine in model animals, but pre-treatment with VPA attenuated the damage due to migraine attack in brain tissues. The level of lipid peroxidation was significantly reduced while the prodcution of anti-oxidant factors was restored. Furthermore, expressions of CGRP and c-Fos, which represented the neuronal activation, were also down-regulated by VPA. The results of western blotting and EMSA demonstrated that the above mentioned effect of VPA acted through the inhibition of NF-кB pathway.

**Conclusions:**

Although controversies on the effect of VPA on NF-кB pathway existed, our study revealed an alternative mechanism of VPA in protecting against migraine, which would promote the development of therapeutic strategies of migraine.

## Background

As a complicated nervous system disease, migraine is characterized by the disability of components in the trigeminal pain pathway. Patients with migraine are attacked by disabling headache associated with sensitivity to afferent inputs, including gastrointestinal inputs, light, sound, and head movement [1]. Based on the investigation in 2002, over 20 % of the world population are affected by migraine at some stage of their entire lives [2], casting severe healthy and social issues to the public health. Although the pathophysiological mechanism which drives the onset of migraine remains poorly understood, compelling evidence infers that nitric oxide (NO) plays a critical role in the pathogenesis of migraine [3–6]. The genes related to NO pathway, including genes encoding endothelial NO synthase (eNOS), inducible NO synthase (iNOS), and vascular endothelial growth factor (VEGF) all increased patients’ susceptibility to migraine [3]. The pathways involved in the induction of migraine by NO may depend on the activation of NF-кB [7], associated with the up-regulation of some key molecules in neuronal activation, i.e., c-Fos [7, 8]. In the normal state, NF-кB is mainly located within the cytoplasm and the subunits of NF-κB, p50 and p65, are complexed with IκBα in an inactive form. Upon stimulation, IκBα is degraded and free NF-κB subunits to enter the nucleus and subsequently cause the expression of inflammatory genes which are the main regulatory enzymes for NO [9]. Thus, some therapies targeting these relevant pathways have been widely investigated regarding their potential to attenuate the damage caused by migraine [10, 11].

Among types of anti-migraine therapies, the protective function of valproate (VPA) against migraine with mild side effects has drawn lots of attention [12]. VPA is achieved by reacting valproic acid (2-Propylpentanoic acid) with a base such as sodium hydroxide and rapidly absorbed in human bodies. VPA reaches peak plasma concentration within one to four hours and thereafter maintains the concentration for four to 14 hours [12]. In clinic, VPA has been widely used as a treatment for seizures and bipolar disorder. Additionally, in many animal models, VPA has improving effect on symptoms associated with stroke, amyotrophic lateral sclerosis, Parkinson’s disease, and Alzheimer’s disease [13–16]. Regarding migraine, researchers in Cochrane Collaboration have affirmed the protective effect of VPA against this disorder in their review in 2015 [12]. However, even with this solid evidence that verifies the potential of VPA in antagonizing migraine, few studies have explicitly revealed the pathways which may be involved in this treatment process. Therefore, the underlying mechanism which drives the action of VPA on migraine remains poorly explained.

In this study, a rat migraine model was established via intraperitoneal injection of nitroglycerin (NTG). As a donor of NO, NTG has been successfully used as an experimental agent to induce migraine in many studies [3, 17, 18]. Administration of NTG can induce activation of cGMP and NF-кB, and further lead to pathogenesis of migraine [6, 19]. The electroencephalogram (EEG) of the experimental animals and the synthesis of oxidative stress molecules which are involved in numerous neurological diseases [20] were detected to assess the effect of VPA on the brain function. The expression of calcitonin gene-related peptide (CGRP) which is closely related to the migraine disorder and headache generation [21], and c-Fos were measured using ELISA, immunohistochemistry, and western blotting assay, respectively. To further explain the pathways involved in the treatment of VPA, the activities of IκBα and NF-κB were determined with western blotting assay and electrophoretic mobility shift assay (EMSA). We hoped that our findings in the present study would provide a preliminary explanation on the mechanism of VPA against migraine and help to promote the application of VPA in clinic.

## Methods

### Chemicals and animals

VPA was purchased from Melonepharma (Catal. No. MB1627, Dalian, China) and dissolved in saline. NTG was purchased from Yimin Pharmaceutical Co., Ltd (Catal. No. H11021022, Beijing, China). Antibodies against c-Fos, p65, IкBα, phosphorylated IкBα (p-IкBα), and Histone H3 were purchased from Beijing Biosynthesis Biotechnology Co., LTD (Catal. No. bs-10172R, bs-5515R, bs-1287R, bs-17422R, Beijing, China). Antibody against NF-кB subunit p65 was purchased from Boster (Catal. No. BA0610. China). Antibody against β-actin was purchased from Santa Cruz Biotechnology, Inc. (Catal. No. sc-47778. USA). Vitamin A standard was purchased from Sigma-Aldrich Co. (Catal. No. R7632, St. Louis, MO, USA). Male Sprague-Dawley rats (weighting *ca.* 200 g) were provided by Experimental Animal Center of China Medical University. All the animals were housed at 20-25 °C with humidity of 55 ± 5 % and had free access to food and water before experimental use. The animal experiments were conducted in accordance with the Institutional Animal Ethics Committee and Animal Care Guidelines of The First Affiliated Hospital of Harbin Medical University.

### Migraine model establishment and pre-administration of VPA

Fifty SD rats were randomly divided into five groups (ten for each group): A) control group, SD rats received an intraperitoneal injection of saline. B) VPAH group, SD rats received intraperitoneal injection of 200 mg/(kg body weight) VPA for five days; C) NTG group, SD rats received intraperitoneal injection of vehicle of VPA each day for five days followed by intraperitoneall injection of 10 mg/(kg body weight) NTG. D) NTG-VPAL group, SD rats received intraperitoneal injection of 100 mg/(kg body weight) VPA for five days followed by injection of 10 mg/(kg body weight) NTG. E) NTG-VPAH group, SD rats received intraperitoneal injection of 200 mg/(kg body weight) VPA for five days followed by injection of 10 mg/(kg body weight) NTG. Four hours after NTG injection, three rats were randomly selected from each group for electroencephalogram (EEG) recording and the left animals were sacrificed for sampling of peripheral blood in jugular vessel and brain tissues.

### Determination of the effect of VPA pre-treatment on the oxidative stress response in brain tissues

The lipid peroxidation was measured with the thiobarbituric-acid reaction with brain homogenate samples, which was determined by comparing the absorption to the standard curve of malondialdehyde (MDA) according to the method proposed by Placer et al. [22] using MDA detection kit according to the manufacturers’ instruction (Catal. No. A003-1, Nanjing Jiangcheng Bioengineering Institute, Nanjing, China). Additionally, the activities of glutathione (GSH) and glutathione peroxidase (GSH-x) in brain homogenate samples were measured according to the manufacturers’ introductions of the assay kits (Catal. No. A006-2, A005, Nanjing Jiancheng Bioengineering Institute, China). Moreover, the concentrations of vitamin A in brain homogenate samples were quantified according to the previously described methods [23–25]. Level of vitamin C and vitamin E in brain homogenate samples were measured using detection kits according to the manufacturers’ introduction (Catal. No. A009, A008, Nanjing Jiangcheng Bioengineering Institute, Nanjing, China).

### Enzyme-linked immuno sorbent assay

The level of CGRP in jugular blood [26, 27] was determined with ELISA method using CGRP detection kit (Catal. No. CEA876Ra USCN, China) according to the manufacturer’s instructions. For jugular blood collection, 1 mL blood samples in each group was drawn from the jugular vessel and stored in Eppendorf tubes containing EDTA (1 mg/ml blood) and the protease inhibitor Aprotinin (0.55 TIU/ml blood).

### Immunohistochemical detection

For immunohistochemical assay, sections were made from trigeminal nucleus caudalis (TNC) tissues from different groups and incubated at 60 °C overnight before dewaxed with dimethylbenzene. The slides were hydrated with alcohol followed by washed with H_2_O_2_ for 5 min, fixed using methanol solution with 3 % H_2_O_2_, and blocked with 1 % goat serum for 15 min at room temperature. They were then incubated with primary anti-c-Fos antibody (1:200) at 37 °C for 30 min before incubated at 4 °C overnight. After four cycles of 0.01 M PBS wash, 5 min for each cycle, secondary antibody (1:200) was added to the slides and placed at 37 °C for 30 min before another four cycles of PBS wash. Slides were incubated with HRP at 37 °C for 30 min before three cycles of 5-min PBS washing. Then DAB was added to the slides and reacted for 3–10 min until the reaction was stopped by ddH_2_O. Slides were re-stained using haematoxylin and dehydrated. Percentage of positively stained cells and the staining intensity of the different groups were determined by observation under a microscope at 400× magnification by experimenters blind to the experiments.

### Western blotting assay

The protein product of c-Fos, IκBα, and p-IκBα in TNC of different groups was extracted using the Total Protein Extraction Kit according to the manufacturer’s instructions (Catalog No. WLA019, Wanleibio, China). Nuclear NF-кB subunit p65 was extracted using Nuclear and Cytoplasm Protein Extraction kit (Catal. No. WLA020, Wanleibio, China). β-actin and Histone H3 were used as internal reference proteins for different molecules. Concentrations of protein samples were determined using the BCA method and western blotting was performed as described previously with some modification [10]: 40 μg protein in 20 μL volume was subjected to 10 % sodium dodecylsulfate polyacrylamide gel electrophoresis (SDS-PAGE). After transferring the proteins onto polyvinylidene difluoride (PVDF) membranes, the membranes were washed with TTBS for 5 min and then incubated with skim milk powder solution for 1 h. Primary antibody against c-Fos (1:500), IκBα (1:500), p-IκBα (1:500), and nuclear NF-кB subunit p65 (1:400), β-actin (1:1000) or Histone H3 (1:500) was added and the membranes were incubated at 4 °C overnight. Following washing with TTBS, the membranes were incubated with HRP-conjugated IgG secondary antibodies (1:5000) for 45 min at 37 °C. After washing, the blots were developed using Beyo ECL Plus reagent and scanned in the Gel Imaging System. The relative expression levels of the target proteins were calculated with Gel-Pro-Analyzer (Media Cybernetics, USA).

### Electrophoretic Mobility Shift Assay (EMSA)

The nuclear protein in TNC samples were extracted using Nuclear and Cytoplasm Protein Extraction kit (Catal. No. WLA020, Wanleibio, China). The DNA binding ability of NF-кB was quantified by EMSA using NF-кB EMSA kit (Viagene, China) according to the manufacturer’s instruction: briefly, the TNC samples were diluted using PBS into 5 μg/μL and subjected to the reaction solution before addition of biotin-labelled NF-кB probe. The blots were developed using Beyo ECL Plus reagent and the results were detected in the Gel Imaging System.

### Statistical analysis

All the data were expressed in the form of mean ± SD and n number for each analysis for each group was five. Post-doc multiple comparisons were conducted by LSD (least significant difference) method using general liner model with a significant level of 0.05. All the statistical analysis and graph manipulation were conducted using R language version 3.2.1 [28].

## Results

### Results of EEG recording

**Fig. 1:**
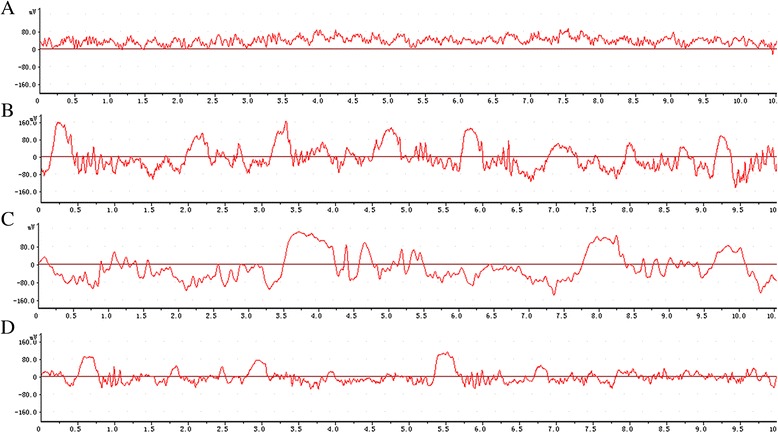
Effect of VPA on electroencephalogram records in brain of migraine-induced rats. **a** image representation of electroencephalogram in control group; **b** image representation of electroencephalogram in NTG group; **c** image representation of electroencephalogram in NTG + VPAL group; **d** image representation of electroencephalogram in NTG + VPAH group

Rats in control group had normal baseline EEGs without any evidence of paroxysmal activity (Fig. 1a). The injection of NTG induced an increase of theta and delta activity in EEG record (Fig. 1b). After administration of VPA, the increase of the slow activity was restored to relatively normal pattern. Moreover, the effect of VPA on NTG-induced migraine was dose-dependent with the EEG in VPAH group performing better than that in VPAL group (Fig. 1c and Fig. 1d). However, there was no significant change in the amplitude of the EEG waves in the four groups. Although our results didn’t provide solid evidence in differentiating migraine from non-migraine cases, it might suggest some difference in the posterior background activity in the EEG in migraine rats as compared to the controls.

### Pre-administration of VPA improved the oxidative stress response induced by NTG injection

Detecting results of MDA, GSH, GSH-x, vitamin A, vitamin C, and vitamin E were shown in Table 1. It was confirmed that sole treatment of VPA of 200 mk/(kg body weight) had no effect on the expression of the six indicators. MDA was representative of the lipid peroxidation and the level was up-regulated by NTG injection, indicating an increase in the lipid peroxidation in brain tissues. Contrary to the change pattern of MDA, the content of GSH and GSH-x were both reduced by NTG injection (Table 1). GSH is the most abundant thiol andioxidant in mammalian cells and maintains the thiol redox in the cells. GSH-x is critical for the reduction of hydro- and organic peroxides with the presence of GSH. The production of three indicators was restored to relatively normal levels in the VPA pre-treated groups, representing the anti-oxidation effect of VPA on brain tissues. The effect of VPA on NTG-induced dys-expression of the three indicators was dose-dependent but there was no significant difference between NTG and NTG-VPAL groups, and only the difference between NTG group and NTG-VPAH group was statistically significant (*P* < 0.05). The change patterns of the content of vitamin A, vitamin C, and vitamin E in different groups were similar to those of GSH and GSH-x (Table 1), which was also indicative of the anti-oxidative potential of VPA as well.

**Table 1:** The effect of VPA on the oxidative stress response in NTG-induced migraine rats (mean ± SD)

Indicators	Group				
	Control	VPAH	NTG	NTG-VPAL	NTG-VPAH
MDA (nmol/mg protein)	10.8 ± 1.9	8.5 ± 2.8	25.3 ± 6.7*^,^**	21.8 ± 4.2*^,^**	13.5 ± 3.5***^,^****
GSH (nmol/mg protein)	8.9 ± 1.2	8.4 ± 1.9	5.2 ± 0.9*^,^**	6.2 ± 1.5*	8.2 ± 1.5***
GSH-x (U/g protein)	17.8 ± 0.6	16.3 ± 3.4	7.8 ± 2.0*^,^**	10.8 ± 3.0*^,^**	15.1 ± 2.1***^,^****
Vitamin A (μmol/g brain)	3.3 ± 0.7	3.2 ± 0.5	1.6 ± 0.3*^,^**	2.1 ± 0.3*^,^**	2.9 ± 0.4***^,^****
Vitamin C (μmol/g brain)	71.0 ± 16.6	69.2 ± 11.7	33.8 ± 8.0*^,^**	44.0 ± 6.6*^,^**	62.5 ± 9.6***
Vitamin E (μmol/g brain)	11.0 ± 1.5	11.8 ± 2.0	5.8 ± 1.5*^,^**	7.4 ± 1.1*^,^**	9.0 ± 1.6***

“*”, significantly different from the control group, *P* < 0.05; “**”, significantly different from the VPAH group, *P* < 0.05; “***”, significantly different from the NTG group, *P* < 0.05; “****”, significantly different from the NTG-VPAL group, *P* < 0.05

### Pre-administration of VPA inhibited the increase of CGRP level in jugular blood and reduced the number of c-Fos positive neurons in TNC

**Fig. 2:**
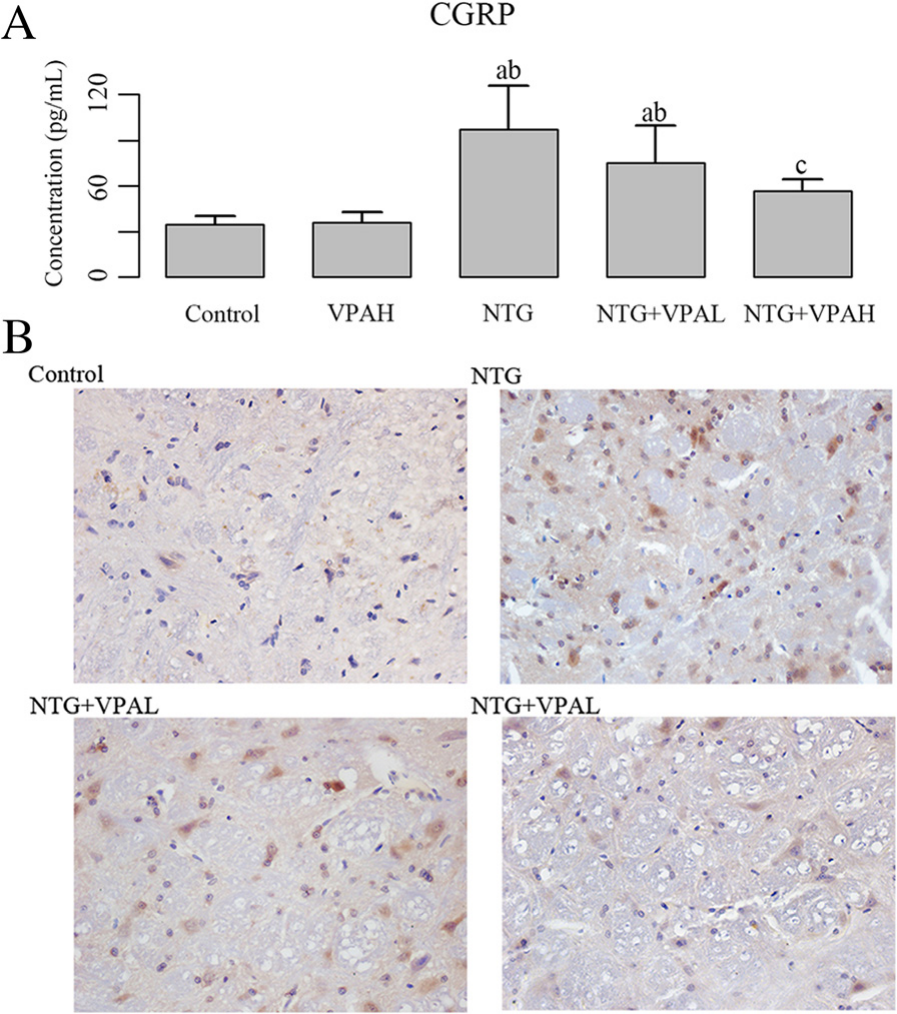
Pre-administration of VPA reduced the expression of CGRP in jugular blood and the expression of c-Fos in trigeminal nucleus caudalis. **a** quantitative analysis results of expression of CGRP in different groups. “a”, significantly different from control group, *P* < 0.05; “b”, significantly different from VPAH group, *P* < 0.05; “c”, significantly different from NTG group, *P* < 0.05. **b** representative images of immunohistochemical staining of c-Fos in different groups, c-Fos positive neurons were stained brown

**Fig. 3:**
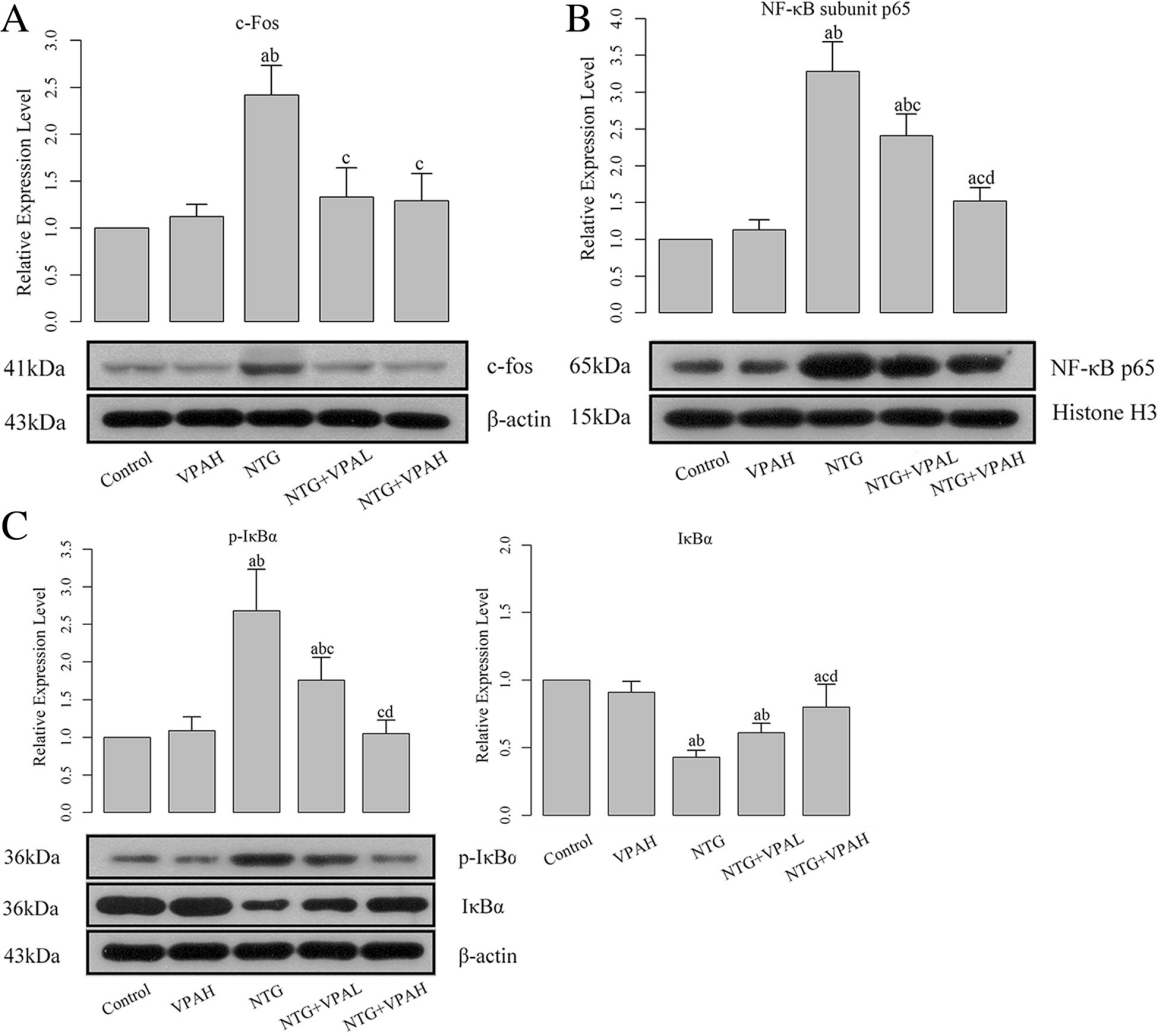
Pre-administration of VPA inhibited the activation of NF-кB pathway. **a** quantitative analysis results and representative images of western blotting of c-Fos; **b** quantitative analysis results and representative images of western blotting of NF-кB subunit p65; **c** quantitative analyses results and representative images of western blotting of p-IкBα and IкBα. “a”, significantly different from control group, *P* < 0.05; “b”, significantly different from VPAH group, *P* < 0.05; “c”, significantly different from NTG group, *P* < 0.05. “d”, significantly different from VPAL group, *P* < 0.05

As show in Fig. 2a, the synthesis of CGRP was also induced by NTG injection, but pre-administration of VPA suppressed the increase and the effect was dose-dependent: the inhibiting effect of 200 mg/(kg body weight) VPA on CGRP was stronger than that of 100 mg/(kg body weight) dose, but only the level of CGRP in NTG + VPAH group was significantly different from that of NTG group (*P* < 0.05). The expression of c-Fos in TNC was assessed using immunohistochemistry (Fig. 2b). c-Fos positive neurons were determined as the brown granules labeled in the cell nucleus. The NTG injection increased the number of c-Fos positive neurons. In VPA pre-treated groups, the densities of brown granules obviously reduced under the observation with a microscope at 400× magnification. Additionally, the expression of c-Fos production illustrated by western blotting exhibited a similar pattern to that of immunohistochemistry (Fig. 3a), and the levels of c-Fos in both NTG + VPAL and NTG + VPAH groups were significantly different from that of NTG group (*P* < 0.05).

### VPA protect brain tissue against the NTG-induced migraine via the inhibition of NF-кB pathway

**Fig. 4:**
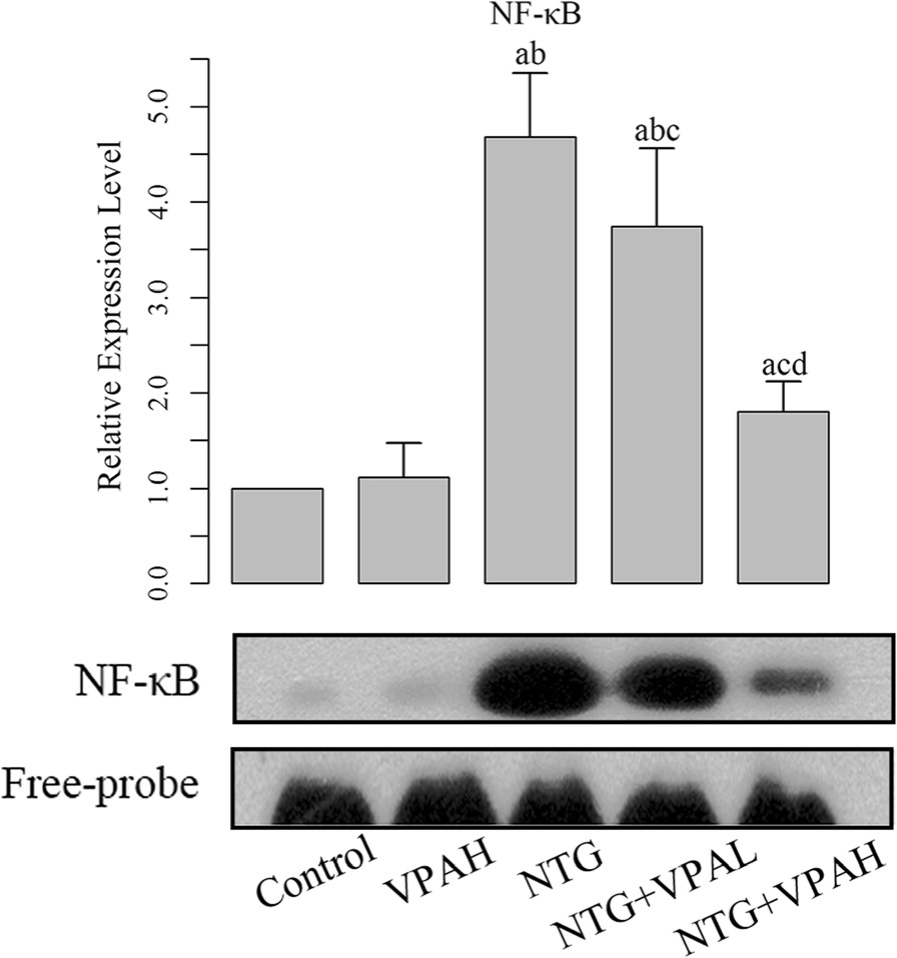
Pre-administration of VPA inhibited the DNA biding ability of NF-кB. “a”, significantly different from control group, *P* < 0.05; “b”, significantly different from VPAH group, *P* < 0.05; “c”, significantly different from NTG group, *P* < 0.05. “d”, significantly different from VPAL group, *P* < 0.05

The expressions of protein product of IκBα, p-IκBα, and NF-кB subunit p65 were quantified using western blotting assay. Production of p-IκBα and nuclear NF-кB subunit p65 was closely related to the activation of NF-кB pathway. After the injection of NTG, it was found the levels of these two molecules were up-regulated (Fig. 3b and 3c), and the differences between control groups and NTG groups were statistically significant (*P* < 0.05). The activation of NF-кB pathway was also verified by the increase of NF-кB’s DNA binding ability detected by EMSA (Fig. 4). Opposite to the expression of p-IκBα and p65, the expression of IκBα was inhibited by NTG injection. IκBα in cytoplasm would bind to NF-кB and inhibit its activation. Our results confirmed that NTG-induced migraine could be initiated by the activation of the NF-кB pathway. Then pre-administration of VPA reversed the expression pattern of NF-кB pathway members due to migraine attack. Both doses of VPA significantly reduced the expressions of p-IκBα and NF-кB subunit p65 (Fig. 3b and 3c), and corresponding to the decreased level of p65, the DNA binding activity of NF-кB was also inhibited (Fig. 4). In addition, the level of cytoplasmic IκBα was restored by pre-administration of VPA. The effect of VPA on the NF-кB pathway was dose-dependent as well, and the difference between the two doses of VPA was statistically significant (*P* < 0.05).

## Discussion

Trigeminovascular system is believed to play a crucial role in the attacks of migraine in classical neuroscience field [29]. The signal that will finally lead to migraine is transmitted to the ventroposterior thalamus from the so-called trigeminocervical complex (or TNC) [30]. In addition, central sensitization associated with activation of NF-кB in the TNC is reported to be involved in the pathogenesis of migraine as well [11]. NF-кB is believed to be implicated in multiple signaling pathways in variable physiological and pathological settings [31], and numerous therapies targeting the NF-кB pathways has been developed to protect patients against migraine [7, 11]. The results of the current study showed that pre-administration of VPA alleviated the impairments due to NTG-induced migraine by inhibiting the activation of NF-кB signal transduction pathway.

Experimental models of migraine induced by NTG injection reflect the inflammatory response with the contribution to the activation of iNOS and cytokines at the meningeal level [19]. Moreover, NTG is evidently proved to trigger the activation of NF-кB in the TNC [7]. In our findings, after injection of NTG, the level of MDA in NTG group was significantly up-regulated compared with control group. It is believed that oxidative stress-induced lipid peroxidation is involved in numerous neurological diseases including migraine [20]. Associated with the increase of lipid peroxidation, significant decrease in antioxidant effectors was also recorded. For GSH and GSH-x, down-regulated of these two molecules in NTG group represented an overload production of reactive oxygen species (ROS) due to NTG-induced migraine attack Also, levels of vitamin A, vitamin E, and vitamin C, which are capable of inhibiting mitochondria-induced ROS production [32], were also significantly inhibited in the brain tissues The low levels of antioxidant vitamins resulted from NTG injection would facilitate oxidative damage in the experimental rats. All the expression patterns of the above mentioned molecules were reversed by the pre-treatment of VPA, evidently indicating the anti-oxidant ability of VPA in migraine. Interestingly, the antioxidant effect of VPA has been found in other research models, including idiopathic epilepsy and primary cultured rat cerebral cortical cells [33–35]. Therefore, we speculated that the protective role of VPA in NTG-induced migraine might be associated with its alleviative effects on oxidative stress damages instead of inhibition of trigeminal neuronal firing.

The migraine antagonizing capability of VPA was also verified by the decrease of jugular blood CGRP which inhibits the synaptic release of various neurotransmitters and is closely related headache generation [26, 36]. Furthermore, the decrease of c-Fos in TNC also supported antioxidant effect of VPA. c-Fos is a sensitive marker of neuronal activation following noxious stimulation, and expression of c-Fos has been widely used to identify areas of neuronal activation and to study neural correlates of nociception as well [37]. As being reported previously, VPA has an inducing effect on g-aminobutyric acid (GABA) in migraine [38]. GABA is a neutral amino acid which binds to one of at least 2 receptor subtypes [39, 40]. Metabolism of GABA is disordered during the attack of migraine [41]. Previous study indicates that administration of VPA restores brain GABA levels and suppresses migraine related events in the cortex [42]. The induction in GABA by VPA results in the inhibition of neuron activation [43], which might affect CGRP and c-fos expression via central and/or peripheral sites of action. However, more than a single GABAergic mechanism might influence the expression of CGRP and c-fos. Thus, experiments targeting GABA based on neural progenitor cells are prepared in our lab to comprehensively explore the mechanism through which VPA exerts its function on CGRP and c-Fos.

The findings in the current study strongly suggested the inductive effect of NTG on NF-кB. NF-кB is a transcriptional factor that regulates the apoptosis and inflammation in various diseases [31]. The inactive form of NF-кB is sequestered in the cytoplasm by binding with IκBα. Once IκBα is phosphorylated into p-IκBα, NF-кB subunits will be translocated into nucleus and activate the transcriptional of the targeted genes. After being treated with NTG, the expressions of p-IκBα and nuclear NF-кB subunit p65 were all enhanced and further led to the activation of NF-кB related pathways. To the opposite, level of IκBα was reduced, which would further result in the release of active form of NF-кB in cytoplasm. Combined with the results of ELISA and immunohitochemical detection, our study was in consistence with the emerging studies that report the activity of NF-кB in regulating the plasticity of neurons and synapsis [31, 44]. In the work of Yin et al., the authors suggest that atorvastatin attenuated the NTG-induced NF-кB activation in TNC in a dose-dependent manner [11]. Furthermore, in the study of Reuter et al., the results infer that NTG promotes NF-кB activity and inflammation, and administration of parthenolide can alleviate the impairment by deactivating the activation of NF-кB and expression of iNOS [45]. Regarding the effect of VPA on NF-кB pathway, Go et al. [46] reported that VPA possesses the ability to inhibit neural progenitor cell death via the activation of NF-кB. However, the results of Go’s study is exactly contrary to ours which indicated that pre-treatment with VPA significantly attenuated the activity of NF-кB pathways and led to the protective effect on nervous system. Although many studies suggest that VPA inhibits NF-кB activity possibly by increasing acetylation on NF-кB [46], at least one study reports that VPA protects neuron from oxidative stress-induced cell death by acetylation-induced activation of NF-кB. Additionally, Rao and his colleague inferred that VPA could inhibit the activation of NF-кB by decreasing the p50 protein levels and had no effect on the phosphorylation state of IкBα, level of total IкBα protein, or protein level of cytosolic p65 in cytosol [47]. But in the current study, solid evidence showed that VPA influenced the activity of both IкBα and p65. These contradictions of the effect of VPA on the NF-кB pathway has been raised by several other studies as well [48–51], which suggest the possibility that the differential regulating pattern of VPA on NF-кB activity in different experimental conditions may be due to the dose- and time window-sensitive characteristics of VPA. Thus, the application of the VPA to modulate NF-кB related pathways should be carefully assessed before being brought into practice.

## Conclusions

In summary, our current work investigated the potential of VPA as a therapy against migraine. Based on a NTG-induced migraine model, it was found that pre-administration with VPA would alleviate the damage due to migraine attack. Through the inhibition of NF-кB pathway in TNC, VPA could reduce the production of ROS in brain tissues. However, the explicit mechanism of VPA acting on NF-кB pathway is still complex. Given the contrary conclusions of ours and previous studies [48–51], application of VPA in clinic should be carefully assessed based on certain conditions. What can be told is that defining the role of VPA in protecting against diseases such as migraine will provide more insights into the pathogenesis and therapeutic strategies of nervous system disorders.
